# Fusion genes in gynecologic tumors: the occurrence, molecular mechanism and prospect for therapy

**DOI:** 10.1038/s41419-021-04065-0

**Published:** 2021-08-11

**Authors:** Bingfeng Lu, Ruqi Jiang, Bumin Xie, Wu Wu, Yang Zhao

**Affiliations:** Department of Obstetrics and Gynecology, Department of Gynecologic Oncology Research Office, Key Laboratory for Major Obstetric Diseases of Guangdong Province, The Third Affiliated Hospital of Guangzhou Medical University, Guangzhou, China

**Keywords:** Cancer genomics, Targeted therapies

## Abstract

Gene fusions are thought to be driver mutations in multiple cancers and are an important factor for poor patient prognosis. Most of them appear in specific cancers, thus satisfactory strategies can be developed for the precise treatment of these types of cancer. Currently, there are few targeted drugs to treat gynecologic tumors, and patients with gynecologic cancer often have a poor prognosis because of tumor progression or recurrence. With the application of massively parallel sequencing, a large number of fusion genes have been discovered in gynecologic tumors, and some fusions have been confirmed to be involved in the biological process of tumor progression. To this end, the present article reviews the current research status of all confirmed fusion genes in gynecologic tumors, including their rearrangement mechanism and frequency in ovarian cancer, endometrial cancer, endometrial stromal sarcoma, and other types of uterine tumors. We also describe the mechanisms by which fusion genes are generated and their oncogenic mechanism. Finally, we discuss the prospect of fusion genes as therapeutic targets in gynecologic tumors.

## Facts

- Fusion genes are cancer-specific and considered to be the driving events of cancer.
- Chromosome instability and genome reassembly are the structural basis for fusion genes.
- Cancer-related exposure factors are closely related to the occurrence of fusion genes.
- Fusion genes change the biological behavior of cancer cells through their molecular functions.

## Open questions

- What is the cause of fusion genes?
- How fusion genes are involved in tumorigenesis and what are the molecular mechanisms?
- What are the characteristics of recurrent fusions in gynecological tumors?
- Are there carcinogenic fusions in gynecological tumors?
- What is the potential of fusion genes in gynecological tumors as therapeutic targets?

## Background

**Fig. 1:**
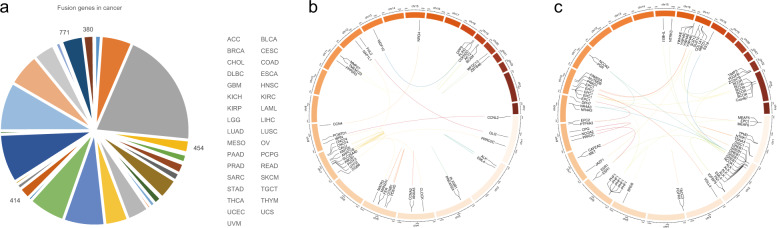
Gene fusions in gynecological tumors. The pie chart shows the number of gene fusions in different types of cancer (**a**). The data comes from the Tumorfusion database. The circos diagram shows the fusion gene on the chromosome in gynecological tumors (**b**), (**c**). Bioinformatic analysis was performed using the OmicStudio tools at https://www.omicstudio.cn/tool.

Fusion genes can be defined as new genes that are formed by chromosome breakage and re-splicing at the genome level [1]. Generally, at the genome level, the fusion gene may be expressed; however, if the promoter region or other important elements are destroyed, it may not be expressed. In 1973, researchers first discovered the rearrangement of chromosomes 9 and 22 in chronic myeloid leukemia (CML) and the rearrangement of chromosomes 8 and 21 in acute myeloid leukemia (AML) through chromosome banding technology [2, 3]. Subsequently, researchers carried out cytogenetic analysis of other hematological tumors, and discovered a variety of cancer-characteristic gene rearrangements. For example, t(8;14)(q24; q32), t(2;8)(p11;q24), and t(8;22)(q24;q11) in Burkitt’s lymphoma [4, 5]; t(4;11)(q21;q23) in acute lymphoblastic leukemia (ALL) [6]; t (15;17) (q22;q21) in acute promyelocytic leukemia (APL) [7]; and t(14;18)(q32;q21) in follicular lymphoma [8]. In recent years, deep sequencing technology has been used widely used, and more cancer-related fusion genes have been characterized. Currently, the identification of fusion genes can be based on whole-genome sequencing (WGS), transcriptome sequencing (RNA-seq), or a combination of the two technologies [9, 10]. Fusion genes identified using WGS alone can be determined to be caused by the rearrangement at the genome level; however, if there is no transcriptome sequencing data, it is impossible to accurately determine whether the new fusion gene is expressed or its expression level. Fusion genes identified by RNA-seq alone can be determined to be expressed (Fig. 1a) [11], but it cannot be completely determined whether this is caused by a genomic mutation or RNA fusion that occurs after the transcription of different genes. Therefore, combining the technology of WGS and RNA-seq can obtain more accurate results. For further verification and analysis, methods such as designing specific primers for PCR or real-time fluorescent quantitative PCR followed by DNA gel electrophoresis can be adopted.

Gene fusion is considered an important driving event for multiple cancers [12]. Some cancer-specific fusion events can be used as diagnostic markers or therapeutic targets, and have achieved good results. In recent years, a large number of fusion events have been described in gynecologic tumors (Fig. 1b, c) [13]; however, their potential roles are not fully understood. Moreover, gene fusion profiles vary in different types of gynecologic tumors. Understanding their occurrence and carcinogenic mechanism will help to identify new therapeutic targets for gynecologic tumors. Therefore, the present article summarizes and classifies the research progress on fusion genes in gynecologic tumors.

## Generation mechanism of fusion genes

### The structural basis of fusion genes

Chromosome rearrangements and extensive mutations in the genome are significant features of cancer [14, 15]. Fusion events are usually caused by chromosomal rearrangements, including translocations, inversions, and deletions [16, 17], and some fusions are caused by cis or trans splicing of adjacent genes transcripts [18, 19]. In the past, it was generally believed that chromosome instability and chromosome rearrangement were the basis of gene fusion, accompanied by breakage at certain specific sites. When the breakpoint is located in the intron region or the exon boundary, a fusion transcript of the complete exon can be retained; however, when the breakpoint is located inside an exon, the corresponding transcript may be destroyed, leading to changes in gene expression profiles [20].

### Physical, chemical, and biological exposure factors induce fusion genes

**Fig. 2:**
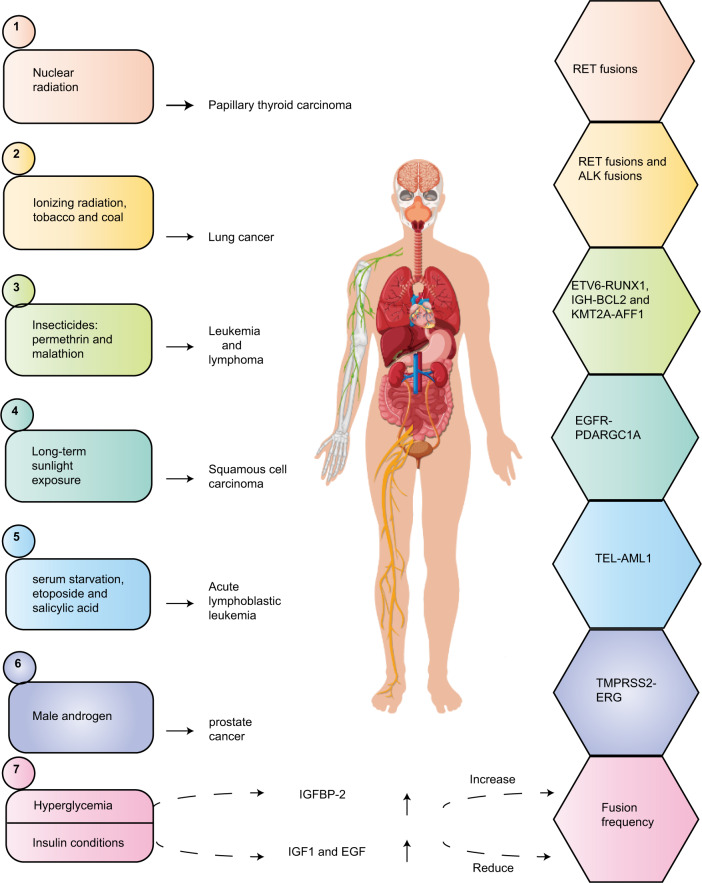
Cancer-related exposure factors induce the corresponding fusion genes. Here, we summarize the fusion genes and their exposure factors in several common cancers, including nuclear radiation and RET fusions; ionizing radiation, tobacco, coal and RETfusions, ALK fusions; insecticides, permethrin, malathion and ETV6-RUNX1, IGH-BCL2, KMN2A-AFF1; long-term sunlight exposure and EGFR-PDARGC1A; serum starvation, etoposide, salicylic acid, and TEL-AML1; male androgen and TMPRSS1-ERG; The relationship between fusion frequency and hyperglycemia/insulin conditions.

Exposure to physical, chemical, and biological factors can cause mutations in the genome and induce gene fusion (Fig. 2). After the Chernobyl nuclear leak, many studies reported that radiation exposure has a strong correlation with gene mutation and gene fusion in thyroid cancer. The frequency of RET fusions in radiation-induced papillary thyroid carcinoma (PTC) is very high, ranging from 35 to 80% [21–23]. A meta-analysis of the distribution of NCOA4-RET, including 2395 cases of radioactive and sporadic PTC, found that radiation exposure caused an increased risk of RET/PTC, although this association was limited to the NCOA4-RET subtype in the Western population [24]. In addition, the ETV6-NTRK3 fusion was found in 14.5% of PTC cases caused by the Chernobyl incident, and this fusion is also considered to correlate strongly with radiation exposure [25]. A study on the relationship between ionizing radiation with RET fusion in lung adenocarcinoma found that 201T human lung cells exposed to 1 Gy of gamma rays induced RET fusion, and RET rearrangement was also found in 2 of 37 cases of radiation exposure [26]. Another study on the frequency of gene fusion in lung cancer showed that patients exposed to tobacco and coal had the highest gene fusion frequency, and ALK fusion and total gene rearrangement were closely related to these exposures [27]. A genomic study of small cell lung cancer with complex tobacco exposure identified the tandem replication of CHD7 exons 3–8 and a cell line with PVT1-CHD7 fusion [28]. In a study of the relationship between insecticides and cancer-related gene damage, the ETV6-RUNX1 fusion was detected in peripheral blood mononuclear cells (PBMCs) that were exposed acutely to permethrin. Exposure to permethrin could induce the fusion of ETV6-RUNX1 and IGH-BCL2 in the K562 cell line, while malathion can induce the fusion of KMT2A-AFF1 and ETV6-RUNX1 [29]. In squamous cell carcinoma of the head, the EGFR-PPARGC1A fusion is associated with long-term sunlight exposure [30]. Hyperglycemia can induce IGFBP2, which increases the frequency of gene fusion, along with a decrease in PKC DNA levels, suggesting that they are mediated by changes in the rate of double-strand break repair. By contrast, IGF1 and EGF are induced under insulin conditions, which reduces the incidence of gene fusion [31]. Some apoptotic signals, such as serum starvation, etoposide, and salicylic acid, can induce TEL (ETV6) gene disruption and fusion in immature B lymphocytes. The TEL-AML1 fusion is one of the most common genetic mutations in childhood acute lymphoblastic leukemia [32]. It is generally believed that prostate cancer is related to male androgen levels. In-depth studies have found that androgen signaling can induce the TMPRSS2 and ERG genes at the genome level, and cells exposed to gamma-rays experience DNA double-strand breaks, thereby promoting TMPRSS2-ERG gene fusion [33]. Interestingly, the expression of the TMPRSS2-ERG fusion gene changed the chemical and radio reactivity of androgen-independent prostate cancer cells [34].

In summary, stimulation by physical, chemical, and biological factors might be crucial conditions for the generation of genomic mutations and gene fusion. Understanding the effects of these exposure factors will help to explore the mechanism of fusion genes in tumorigenesis and provide protective strategies in cancer prevention and treatment.

## General mechanism of fusion genes in cancer

**Fig. 3:**
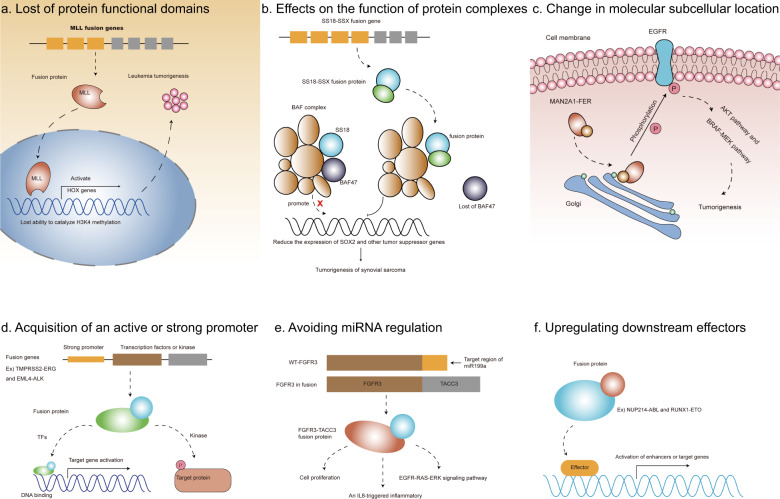
General mechanism of fusion genes in cancer. MLL fusions lost its functional domain which catalyze H3K4 methylation in Leukemia tumorigenesis (**a**). SS18-SSX fusion protein cause the lost of BAF47 subunit of BAF complex in synovial sarcoma (**b**). MAN2A1-FER fusion allows the FER molecule to be located to the Golgi apparatus, thereby activating its tyrosine kinase activity (**c**). Fusion genes allow some transcription factors or kinases to acquire strong promoters, thereby activating downstream genes (**d**). FGFR3-TACC3 fusion avoids the regulation of miR199a in tumorigenesis (**e**). Some fusion proteins act as effectors to activate the target enhancers or genes (**f**).

Gene fusions can result in the production of new fusion transcripts or fusion proteins. Some fusion products play a key driving role in cancer, such as BCR/ABL and AML1/ETO fusions in hematological malignancies [35, 36]. In recent years, many potential molecular functions of oncogenic fusion proteins have been discovered. Here, we summarize the carcinogenic mechanisms of several fusion genes (Fig. 3), including: destroying protein functional domains, affecting the function of protein complexes, changing molecular subcellular localization, obtaining active or strong promoters, evading regulation by microRNAs (miRNAs), and upregulation of downstream effectors.

### Loss of protein functional domains

Some gene fusions result in the loss of part of the coding exons during the rearrangement process, depending on the location of the breakpoint. The loss of important structural domains will cause functional defects (Fig. 3a). The MLL gene encodes a DNA binding protein containing a SET domain, which has H3K4 methyltransferase activity [37]. The MLL gene can regulate positively the expression of a series of downstream HOX genes in hematopoietic stem cells through its H3K4 methyltransferase activity. The fused MLL gene produces a truncated MLL protein lacking the SET domain, resulting in its inability to catalyze H3K4 methylation, which drives the occurrence of leukemia.

### Effects on the function of protein complexes

Functional protein complexes are common, and structural integrity is crucial for their normal function. Repeated chromosomal rearrangement is an important sign of synovial sarcoma. The SS18-SSX fusion produced by t(X;18) has been confirmed to be a carcinogenic fusion in synovial sarcoma (Fig. 3b) [38]. SS18 encodes a subunit of the SWI/SNF (BAF) complex and this gene is often rearranged in synovial sarcoma. The SS18-SSX fusion protein competes with the wild-type SS18 protein for binding to the BAF complex, resulting in the loss of the BAF47 subunit (a tumor suppressor) from the complex, which promoting the expression of downstream SOX2 and other genes [39]. In addition, the synergy of the SS18-SSX fusion protein and SWI/SNF complex plays an important role in chromatin structure regulation and histone modification, which jointly promote the tumorigenesis and development of synovial sarcoma [40].

### Change in molecular subcellular location

The correct subcellular location of a protein is a significant factor for signal transduction and protein molecular function. Some partner genes that express proteins with specific subcellular localization sequences can play a role by changing the localization of the fusion protein (Fig. 3c). MAN2A1-FER is a common recurrent fusion in liver cancer, esophageal adenocarcinoma, and non-small cell lung cancer, and has carcinogenic effects [41, 42]. The fused MAN2A1 protein retains its signal peptide, resulting in the MAN2A1-FER fusion protein being located in the Golgi apparatus, which results in the ectopic activation FER. The FER-related tyrosine kinase activity of the fusion protein is almost four times that of wild-type FER, which activates downstream signaling molecules significantly, such as BRAF, MEK, and AKT.

### Acquisition of an active or strong promoter

The promoter is an important element of gene expression, and its activity is a key factor in determining gene transcription efficiency. It has been reported that some genes acquire active promoters or cis-regulatory elements through fusion events to achieve higher expression levels (Fig. 3d) [43]. For example, the TMPRSS2-ERG fusion is a carcinogenic fusion event in prostate cancer. The fusion allows the ERG gene to share clusters of regulatory elements (COREs) from the TMPRSS2 gene, including its promoter. ERG is as a key transcription factor that maintains the expression of genes required for cancer cell proliferation and metabolism. The EML4-ALK fusion is a successful target for the treatment of non-small cell lung cancer. The fused ALK acquires an active promoter and dimerization site from the EML4 gene, resulting in constitutively activated ALK kinase [44]. Therefore, obtaining a strong promoter is an important mechanism by which oncogenic transcription factors or kinases drive cancer.

### Avoiding miRNA regulation

MicroRNAs are small non-coding RNAs with a length of 18–22 nt. miRNAs target and bind to the 3′ untranslated region (UTR) of their target gene mRNAs, leading to mRNA degradation or translational silencing, which play negative roles in the regulation of gene expression (Fig. 3e) [45]. It has been reported that FGFR3 is the target gene of miR-99a and is negatively regulated by miR-99a. In FGFR3-TACC3 fusion-positive gliomas, the fused FGFR3 loses its 3′ UTR region; therefore, it is no longer negatively regulated by miR-99a, which increases the FGFR3 tyrosine kinase signal, thereby promoting tumor progression [46].

### Upregulating downstream effectors

The enhancer region is important to regulate gene transcription activity, and the silencing or activation of an enhancer is usually mediated by certain transcriptional activation or repressor factors. For example, the transcriptional activator STAT5 activates the transcription of target genes (such as MYC and BCL2) by binding to their enhancer regions and co-exists with enhancer markers such as BRD4, P300, and H3K27ac. The NUP214-ABL fusion is considered to be a key factor in inducing acute T-cell leukemia (Fig. 3f). The fused protein can activate STAT5 through phosphorylation, and activated STAT5 can increase the transcriptional activity of its target genes significantly [47, 48]. In AML, the RUNXI-ETO fusion protein is considered to be an oncogenic transcription factor (Fig. 3f). The RUNX1-ETO fusion protein replaces the wild-type RUNX1 protein and binds to the open chromatin region upstream of the CCND2 transcription start site (TSS), resulting in the abnormal activation of CCND2, which drives the occurrence of AML [49].

## Current research status of fusion genes in gynecologic tumors

The research progress into gene fusions in various types of gynecologic tumors varies. There have been more studies related to ovarian cancer and uterine sarcoma, and fewer studies related to cervical cancer and endometrial cancer. In this section, we describe gene fusions in various types of gynecologic tumors, including the frequency of fusion genes, the method of fusions, the relationship between the genes and cancer, and the mechanism of oncogenic fusion (Table 1). All fusion genes have been confirmed in the relevant literature. The identification methods of fusion genes include RT-PCR, FISH or Sanger sequencing.

**Table 1:** Characteristic of fusion genes in gynecologic oncology.

*Fusion genes*	*Frequency*	*Cancer type*	*Chromosome*	*Junction*	*Molecular mechanism*	*Role in cancer*	*References*
PCMTD1-CCNL2	22% (4/18)	Ovarian cancer	8, 1	3, 6	–	–	[52]
ANXA5-CCNA2	5.6% (1/18)	Ovarian cancer	4, 4	3, 3	–	–	[52]
PDE4D-CCNB1	5.6% (1/18)	Ovarian cancer	5, 5	1, 2	–	–	[52]
SLC25A40-ABCB1	15.7% (20/108)	Ovarian cancer	7, 7	–, 2	Upregulate ABCB1 expression	Associated with drug resistance	[55]
TMEM123-MMP27	–	Ovarian cancer	11, 11	2, 7	Increase the expression of the 3′ end gene	–	[72]
ZBTB46-WFDC13	–	Ovarian cancer	20, 20	–, 2	Increase the expression of the 3′ end gene	–	[72]
PLXNB1-PRKAR2A	–	Ovarian cancer	3, 3	9, 7	Increase the expression of the 3′ end gene	–	[72]
MAN2A1-FER	1.7% (1/60)	Ovarian cancer	5, 5	13, 6	Significantly improve FER tyrosine kinase activity	Oncogenic	[42]
CDKN2D-WDFY2	20% (12/60)	Ovarian cancer	19, 13	1, 13	Express a truncated WDFY2 protein	–	[60]
BCAM-AKT2	7% (4/60)	Ovarian cancer	19, 19	13, 5	Guide the fused AKT2 to the membrane where it is activated by phosphorylation	Oncogenic	[66]
FHL2-GLI2	*65% (17/26)*	Sclerosing stromal tumors of the ovary	2, 2	5, 8	Activate the Sonic hedgehog (Shh) signaling pathway	Oncogenic	[67]
CPQ-PRKDC	2.5% (3/122)	Endometrial cancer	8, 8	2, 80	–	–	[75]
EPC1-SUZ12	–	Endometrial stromal sarcoma	10,17	10, 2	–	–	[85]
EPC1-BCOR	–	Endometrial stromal sarcoma	10, X	7, 11	–	–	[85]
EPC1-PHF1	–	Endometrial stromal sarcoma	10, 6	10, 2	–	–	[86]
MEAF6-PHF1	–	Endometrial stromal sarcoma	1, 6	5, 2	–	–	[92]
BRD8-PHF1	–	Endometrial stromal sarcoma	5, 6	16, 2	–	–	[93]
EPC2-PHF1	–	Endometrial stromal sarcoma	2, 6	13, 2	–	–	[94]
JAZF1-PHF1	–	Endometrial stromal sarcoma	7, 6	2, 1	–	–	[86]
YWHAE-FAM22A	26–58%	Endometrial stromal sarcoma	17, 10	5, 2	Activates the expression of a series of downstream genes, such as CCND1 and CEBPA	Oncogenic	[97, 98]
JAZF1-SUZ12	15–50%	Endometrial stromal sarcoma	7, 17	3, 2	Changes the structure of the PRC2 complex and inhibits its H3K27 methylation activity	Oncogenic	[101, 106]
MBTD1-CXorf67	7%	endometrial stromal sarcoma	17, X	16, 1	Increased the expression of CXorf67	–	[107]
FGFR3-TACC3	1.9% (2/103)	Cervical cancer	4, 4	17, 4&6	Activates the MAPK pathway by increasing its FGFR3 signal	Oncogenic	[80, 81]
IGFBP5-ALK	27% (3/11)	Uterine inflammatory myofibroblastoma	2, 2	1, 19	–	–	[110]
THBS1-ALK	27% (3/11)	Uterine inflammatory myofibroblastoma	15, 2	4, 19	–	–	
FN1-ALK	18% (2/11)	Uterine inflammatory myofibroblastoma	2, 2	–	–	–	
TIMP3-ALK	9% (1/11)	Uterine inflammatory myofibroblastoma	22, 2	1,19&20	–	–	
GREB1-NCOA2	–	UTROSCT	2, 8	3, 14	–	Tumors with GREB1 rearrangements are more aggressive	[113]
GREB1-NR4A3	–	UTROSCT	2, 9	7, 2	–		
GREB1-SS18	–	UTROSCT	2, 18	7, 6	–		
GREB1-NCOA1	–	UTROSCT	2, 2	7, 13	–		

The table shows the frequency, cancer type, chromosome location, the site of the junction, molecular mechanism, and role in cancer of all the confirmed fusion genes.

### Fusion genes in ovarian cancer

Among gynecologic tumors, ovarian cancer is a highly malignant tumor that is prone to metastasis and recurrence. Approximately 70–80% of patients with advanced ovarian cancer experience recurrence within 5 years [50, 51]. The fusion genes that have been identified in ovarian cancer are PCMTD1-CCNL2, ANXA5-CCNA2, CCN4-NRG4, SLC25A40-ABCB1, DPP9-PPP6R3, MAN2A1-FER, CDKN2D-WDFY2, BCAM-AKT2, and FHL2-GLI2. These fusion genes involve cyclin family genes, multi-drug resistance related MDR/TAP subfamily genes, tyrosine kinase family genes, and AKT signaling pathway-related genes [52].

The frequency of the PCMTD1-CCNL2 fusion in endometrioid ovarian cancer is 22% (4/18). It is formed by the rearrangement of the PCMTD1 gene on chromosome 8 and the CCNL2 gene on chromosome 1. The upstream part of the fusion junction is exon 3 of PCMTD1, and the downstream part is exon 6 of CCNL2. The parental gene CCNL2 encodes a cyclin family protein that can interact with a variety of proteins to induce cell cycle arrest and apoptosis in lung cancer and mouse embryonic cancer cells [53, 54]. Interestingly, the other parent gene, PCMTD1, encodes a member of the methyltransferase superfamily, which has methyltransferase activity. Unfortunately, no studies have confirmed that this gene fusion affects the occurrence or development of ovarian cancer or other cancers.

The SLC25A40-ABCB1 fusion is the most common rearrangement involving ABCB1, occurring in 15.7% (20/108) of cases of high-grade serous ovarian cancer [55]. Most of the breakpoints of ABCB1 fusions occur in the intron 1 region, and the fusion includes exon 2 and the following sequence. ABCB1 fusion transcription promoters are mostly replaced by partner gene promoters. ABCB1 fusion-positive tumor tissues are usually accompanied by upregulation of ABCB1 expression, suggesting that gene fusion might allow ABCB1 to escape negative regulation and obtain a strong promoter. The SLC25A40 gene is commonly expressed in tissues and has not been shown to be related to tumors. MDR1, the protein encoded by ABCB1, is a member of the MDR/TAP subfamily, which mediates the efflux of chemotherapeutic drugs and is associated with multidrug resistance in tumors [42, 56].

Although the frequency of MAN2A1-FER fusion in ovarian cancer is only 1.7% (1/60), it appears in a variety of tumors. The frequency of MAN2A1-FER is higher in esophageal adenocarcinoma (25.9%), liver cancer (15.7%), and non-small cell lung cancer (16.8%) [42]. The MAN2A1-FER fusion is formed by the cis splicing of exons 1–13 of MAN2A1 and exons 1–6 of FER on chromosome 5. The parental gene MAN2A1 encodes a glycosyl hydrolase located in the Golgi apparatus that catalyzes the final hydrolysis step in the maturation pathway of asparagine-linked oligosaccharides (N-glycans). MAN2A1 has been shown to be involved in the immune regulation of tumors, and inhibition of MAN2A1 can enhance the tumor’s immune response to anti-PD-L1 drugs [57]. The encoded product of FER is a member of the receptor tyrosine kinase family, which regulates cell adhesion and is related to the epithelial-mesenchymal transition process of tumors [58]. Mechanistically, the MAN2A1-FER fusion protein retains the signal peptide of MAN2A1, which results in the fusion protein being located in the Golgi apparatus, significantly increasing the activity of the fused FER tyrosine kinase [42].

The CDKN2D-WDFY2 fusion is cancer-specific and has only been identified in ovarian cancer. The fusion is formed by the rearrangement of CDKN2D on chromosome 19 and WDFY2 on chromosome 13, including exon 1 of CDKN2D and exons 3–12 of WDFY2, with a fusion frequency of 20% (12/60) [59]. CDKN2D encodes a cell cycle regulator that is involved in the DNA repair process. WDFY2 encodes a protein containing two WD domains and an FYVE zinc finger region, which can specifically target AKT2, leading to decreased phosphorylation of downstream molecules of AKT (such as BAD and FOX3A) [60]. Studies have shown that overexpression of WDFY2 can inhibit the biological behavior of prostate cancer by affecting the AKT pathway [61]. The CDKN2D-WDFY2 fusion resulted in the loss of wild-type WDFY2 protein expression, and the truncated WDFY2 protein was expressed at the same time. The fused WDFY2 protein lacks the AKT-binding domain and results in increased downstream BAD and FOX3A expression.

The BCAM-AKT2 fusion is a specific rearrangement in high-grade serous ovarian cancer. The fusion frequency is 7% (4/60). The fusion gene comprises exons 1–13 of BCAM and exons 5 and the following sequence of AKT2 on chromosome 19. BCAM encodes a receptor for the extracellular matrix protein laminin, which mediates the adhesion of red blood cells [62] and is related to the metastasis of colorectal cancer [63–65]. AKT2 is a known oncogene encoding a serine/threonine kinase subfamily protein that can phosphorylate a variety of proteins. Mechanistically, the BCAM-AKT2 fusion protein uses the membrane localization domain of BCAM to guide the fused AKT2 to the membrane where it is activated by phosphorylation [64, 66].

The FHL2-GLI2 fusion is unique to sclerosing stromal tumors of the ovary (SST), with a fusion frequency of 65% (17/26). It consists of exons 1–5 of FHL2 and exons 8–12 GLI2 on chromosome 2 [67]. FHL2 has been reported to play a role in a variety of tumors, participating in epithelial-mesenchymal transition and stabilizing EGFR [68, 69]. The encoded product of GLI2 is a member of the transcription factor of the Gli family, which participates in the Sonic hedgehog (Shh) signaling pathway and promotes tumor progression [70, 71].

Although the TMEM123-MMP27, ZBTB46-WFDC13, PLXNB1-PRKAR2A, and other fusions have been reported in only a few cases of ovarian cancer, it is interesting that the above fusions increase the expression of the 3′ end gene [72] 5. In addition, a study reported that the expression levels of 48 genes located near the fusion gene were upregulated significantly, which also indicated that fusion gene remodeling of the cancer transcriptome is a complicated process [73].

### Fusion genes in endometrial cancer

The pathogenesis of endometrial cancer is still unclear. Studies have shown that mutations in some genes or pathways are important driving events of uterine corpus endometrial carcinoma (UCEC), such as the mutation of the P53 gene, the PIK3CA pathway, the KRAS gene, and overexpression of the HER2 gene [74]. In recent years, with the development of deep sequencing technology, a large number of fusion events have been discovered (see Table 1), among which CPQ-PRKDC and TSNAX-DISC1 are two representative fusions [75]. CPQ-PRKDC occurs at a low frequency in endometrial cancer (2.5%, 3/122), and is formed by the rearrangement of exons 1–2 of CPQ and exons 80–87 of PRKDC on chromosome 8 [76]. The TSNAX-DISC1 fusion is not a gene-level rearrangement. It is formed by the splicing of TSNAX and DISC1 transcripts and occurs with a frequency of 71.2% (123/176) in UCEC. Unfortunately, there is no research to show whether these two fusions play a role in cancer.

### Fusion genes in cervical cancer

Chronic persistent infection of high-risk human papillomavirus (HPV) is the main cause of cervical cancer, and more than 90% of cases are accompanied by HPV virus infection [77]. However, only 1% of women infected with high-risk HPV eventually develop into cervical cancer. This suggests that there may be other carcinogenic factors besides HPV infections, such as gene mutations and chromosome rearrangement. We found 454 fusions in the Tumorfusion database (Fig. 1a) [11], but only the FGFR3-TACC3 fusion has been confirmed [78]. This fusion is formed by the cis splicing of two adjacent genes on the P16 arm of chromosome 4, including exons 1–17 of FGFR3 and exons 4–16 or 6–16 of TACC3, with an incidence of 1.9% (2/103). FGFR3 encodes a member of the fibroblast growth factor receptor (FGFR) family and contains a tyrosine kinase domain. TACC3 encodes a member of the acidic Escherichia coli chain protein family, which plays a role in the differentiation and growth of certain cancer cells. In glioma, FGFR3 is regulated negatively by miR-99a, while in cervical cancer, FGFR3-TACC3, which has lost the FGFR3 3′ UTR region, has been proven to be a carcinogenic fusion that activates the MAPK pathway by increasing its FGFR3 signal [79]. In addition, the FGFR3-TACC3 fusion protein can replace the EGFR-ERK signaling pathway in tumors, mediating the drug bypass resistance mechanism [80]. This suggests that FGFR3 fusions can fully exert their carcinogenic effects by escaping the negative regulation of miR-99a [81, 82]. Studies have reported that FGFR3-TACC3 is a common fusion in many tumors, and the fusion frequency is similar in various cancers [83]. The FGFR3-TACC3 fusion is a clear oncogene, and its targeted inhibition has achieved good results in other cancers.

### Fusion genes in endometrial stromal sarcoma (ESS)

The cause of ESS is unclear; however, it has a high recurrence rate and poor prognosis. It is generally believed that chromosomal rearrangement is closely related to the occurrence of ESS [84]. The instability of chromosomes 6, 7, 10, and 17 in ESS is the cause of some fusions, such as PHF1, JAZF1, EPC1, YWHAE, and MBTD1-CXorf67 fusions.

EPC1 fusion is a rare event in ESS, being only reported in a few cases. Its partner genes include SUZ12, BCOR, and PHF1. EPC1-PHF1 has been reported very early and is associated with the morphology and clinical features of low-grade ESS, which is produced by t (6;10) (p21; p11). By contrast, EPC1-SUZ12 and EPC1-BCOR are more prone to occur in aggressive high-grade ESS, corresponding to t (10;17) (p11; q11) and t (10; *x*) (p11; p11), respectively [85, 86]. EPC1 is an oncogene that can act as both a transcriptional activator and a repressor, and is related to apoptosis and DNA repair [87, 88]. For example, the EPC1 promoter physically combines with E2F1 to activate transcriptional activity, thereby inducing anti-apoptosis-related genes and promoting cancer growth. Interestingly, the three partner genes are all related to transcriptional regulation [89–91], suggesting that the fusion of EPC1 and its partner genes allows them to be physically close, which might promote its participation in transcriptional regulation.

PHF1 fusions mainly include MEAF6-PHF1, EPC1-PHF1, BRD8-PHF1, EPC2-PHF1, and JAZF1-PHF1 [92–94]. The PHF1 gene is located on chromosome 6 (6p21), JAZF1 is located at 7p15, EPC2 is located at 2q23, and EPC2 is located at 10p11. The rearrangement of EPC1 or MEAF6 from 1p34 is a recurrent fusion in ESS. The encoded product of PHF1 is a polycomb group protein, which forms a PRC2 complex with, for example, MTF2 and PHF19 to mediate the methylation of histone H3K27 [95]. The PRC2 complex is a methyltransferase that is commonly dysregulated in human cancers. Overexpression of PRC2 is a significant sign of poor prognosis in human cancer [96]. Similar to the EPC1 fusion, most of the PHF1 fusion partner genes are also involved in transcriptional regulation, which suggests that fusion genes might be a mechanism of transcription disorders in ESS.

YWHAE-FAM22A/B fusion is a rearrangement of chromosome 17 arm p13 with chromosome 10 arms q23 and q22, including exons 1–5 of YWHAE and exons 2–7 of FAM22A, with a frequency of 26–58% in ESS [97, 98]. Compared with JAZF1 fusions, YWHAE fusions ESS tend to be associated with higher disease stages and more frequent recurrences, and have diagnostic specificity for high-grade ESS. The encoded product of YWHAE is 14-3-3ε, which belongs to the 14-3-3 protein family, and mediates signal transduction by binding to proteins containing phosphoserine residues. For example, the combination of 14-3-3ε and FBX4 promotes the dimerization of FBX4 and promotes its E3 ligase activity [99]. In prostate cancer, the combination of 14-3-3ε and APAF-1 inhibits cytochrome c from activating the downstream caspase and protects the survival of cancer cells [100]. FAM22A/B encodes a protein with a nuclear localization signal; however, its role in cancer is unclear. The YWHAE-FAM22A/B fusion retains the nuclear localization sequence of FAM22A/B and maintains the complete 14-3-3ε domain. YWHAE, which is directed to the nucleus from the cytoplasm, activates the expression of a series of downstream genes, such as CCND1 and CEBPA [98].

JAZF1-SUZ12 is a carcinogenic fusion related to low-grade ESS. Its fusion frequency is reported to be 75% in endometrial stromal nodule (ESN), 50% in low grade (LG)-ESS, and 15% in high grade (HG)-ESS, and can be used to distinguish LG-ESS from HG-ESS [101]. This fusion is the result of a rearrangement between p15 of the arm of chromosome 7 and q21 of the arm of chromosome 17, which contains exons 1–3 of JAZF1 and exons 2–16 of SUZ12. JAZF1 encodes a nuclear protein with a zinc finger structure. This protein binds to the orphan nuclear receptor TAK1 and acts as a transcriptional regulator [102], which can play a role in tumor suppression or cancer promotion [103, 104]. SUZ12 encodes an important component of the PRC2 complex, which mediates the modification of H3K9 and H3K27 methylation, and is related to chromatin remodeling and gene silencing [105]. The JAZF1-SUZ12 fusion protein in ESS replaces the wild-type SUZ12 protein, which changes the structure of the PRC2 complex and inhibits its H3K27 methylation activity, thus increasing the expression of downstream genes HOXA9 and WNT11 [106].

The fusion frequency of MBTD1-CXorf67 in ESS is 7% (1/14). The MBTD1-CXorf67 fusion is composed of exons 1–16 of MBTD1 and exon 1 of CXorf67 [107]. The result of the MBTD1-CXorf67 fusion in ESS increased the expression of CXorf67 by 5–9 times. Although it is not clear whether CXorf67 has an oncogene effect in ESS, studies have confirmed that CXorf67 affects the DNA repair pathway of homologous recombination in the ependymoma and blocks the methyltransferase activity of EZH2, which plays an important role in glioma tumorigenesis.

### Fusion genes in other types of uterine tumors

Uterine inflammatory myofibroblastoma (IMT) is a rare mesenchymal tumor with low-grade malignancy, the recurrence of which can be effectively avoided by complete surgical resection [108]. IMT is usually accompanied by ALK expression and ALK fusion. Common gene fusions in IMT include IGFBP5-ALK, THBS1-ALK, FN1-ALK, and TIMP3-ALK. ALK fusion is considered a diagnostic indicator of IMT [109]. A study found that 10 of 11 IMT tumors contained ALK rearrangements, among which IGFBP5-ALK represented 27% (3/11), THBS1-ALK represented 27% (3/11), FN1-ALK represented 18% (2/11), and TIMP3-ALK represented 9% (1/11) [110]. Another study detected ALK gene fusions in 14 cases of IMT, which indicated that the incidence of ALK fusions in IMT is extremely high [109]. ALK encodes a well-known receptor tyrosine kinase. The ALK fusion partner usually produces oncogenic constitutive tyrosine kinase activity by promoting the polymerization and autophosphorylation of ALK [111].

Uterine tumors similar to ovarian sex cord stromal tumors (UTROSCT) are rare uterine stromal tumors of unknown etiology. Although most of them are benign, some of them recur [112]. Chromosome rearrangement has recently been reported in UTROSCT cases, where ESR1 fusion and GREB1 fusion are common, including ESR1-NCOA2, ESR1-GREB1, GREB1-NCOA2, GREB1-NR4A3, GREB1-SS18, GREB1-NCOA1, and GREB1-CTNNB1. In a recent study, four cases of UTROSCT with GREB1 rearrangements were identified, including GREB1-NCOA2, GREB1-NR4A3, GREB1-SS18, and GREB1-NCOA1. UTROSCTs with these rearrangements were found to be more aggressive than those with ESR1 rearrangements [113]. GREB1 is an early estrogen-responsive gene in the estrogen receptor regulatory pathway. It is believed to stimulate the proliferation of breast, ovarian, and prostate cancer cells [114], and mediate resistance to tamoxifen [115]. NCOA1 and NCOA2 are common partner gene and their encoded proteins act as transcriptional co-activators of steroid and nuclear hormone receptors. They contain nuclear localization signals and bHLH and PAS domains. ESR1 encodes a transcription factor that activates estrogen receptors and ligands, and is considered to be a driving event for breast and endometrial cancer [116, 117]. These reports suggest that fusion genes of the estrogen regulatory pathway have potential carcinogenic effects in UTROSCT.

## The prospect of gene fusions as therapeutic targets in gynecologic tumors

For more than half a century, gene fusion has been regarded as a key driving event in cancer. The results of gene fusion can change the expression pattern of the original gene. Some fusion genes can activate protein functions or produce new chimeric proteins, and can participate in the occurrence and development of cancers by changing signaling pathways, affecting protein interaction, changing subcellular localizations, or activating neighboring genes. Chromosome rearrangement and gene fusions are valuable in the diagnosis or classification of cancers [16]. Studies also suggested that gene fusion can be used as a method to monitor the residual small lesions after treatment [118]. Many cancers characterized by fusion genes have a poor prognosis; therefore, targeting specific oncogenic fusion proteins might bring unexpected results. In recent years, targeted drug therapy for gene fusion-positive cancers has significantly improved the prognosis of patients with cancer. For example, tyrosine kinase inhibitors have achieved satisfactory results in a variety of cancers. With the development of deep sequencing technology, scientists have discovered that fusion genes not only exist in sarcomas and hematological malignancies, but also exist in large amounts in some solid tumors [119, 120]. Although most of the fusion events seem to occur accidentally and have no pathogenic characteristics, there are some valuable fusions among the few commonly recurring fusions.

Some of the fusions described in this article have potential value for the management of gynecologic tumors. For example, the JAZF1-SUZ12 fusion related to low-grade ESS and the YWHAE-FAM22 fusion related to high-grade ESS have been confirmed to play a role in the progression of ESS [98, 106]. The CDKN2D-WDFY2 fusion had a detection rate of 20% in 60 cases of ovarian cancer, and it is not found in normal ovarian and fallopian tube tissues [59]. WDFY2 is an important regulator of AKT phosphorylation, and thus might represent a promising target in the diagnosis and treatment of ovarian cancer. The MAN2A1-FER fusion in ovarian cancer can significantly enhance tyrosine kinase activity. In vivo experiments have also confirmed that the fusion promotes tumor progression. Therefore, the application of tyrosine kinase inhibitors to MAN2A1-FER fusion-positive cancers might be a valuable choice [42]. In cervical cancer, glioma, and other tumors, FGFR3-TACC3 is a recurrent and carcinogenic fusion [46, 79], and is sensitive to FGFR inhibitors [83]; therefore, FGFR3-TACC3 fusion protein inhibitors have the potential to treat fusion-positive cancers. At present, the targeted drugs used in gynecologic tumors mainly include VEGF inhibitors, EGF inhibitors, and PARP inhibitors. Although these drugs have achieved certain results, the rates of recurrence and metastasis are still high.

## Conclusions

A full understanding of the frequency, associated pathways, and carcinogenic mechanisms of fusion genes in gynecologic tumors have great prospects to identify valuable oncogenic fusions and potential targets.

## Data Availability

The data used to support the findings of this study are available from the corresponding author upon request.
